# Supplementary material for: Identification and functional analysis of non-coding regulatory small RNA FenSr3 in Bacillus amyloliquefaciens LPB-18
Source: PeerJ. 2023 May 15;11:e15236. doi: 10.7717/peerj.15236 (PMC10194069; doi:10.7717/peerj.15236)
Supplement: Supplemental Information 4 [file peerj-11-15236-s004.zip › KO/CK-vs-T1_map/map04370.html]

KEGG PATHWAY: VEGF signaling pathway - Reference pathway


|  |  |
| --- | --- |
| **VEGF signaling pathway - Reference pathway** |  |

[
Pathway menu
| Organism menu
| Pathway entry
| Show description
| User data mapping
]

There is now much evidence that VEGFR-2 is the major mediator of VEGF-driven responses in endothelial cells and it is considered to be a crucial signal transducer in both physiologic and pathologic angiogenesis. The binding of VEGF to VEGFR-2 leads to a cascade of different signaling pathways, resulting in the up-regulation of genes involved in mediating the proliferation and migration of endothelial cells and promoting their survival and vascular permeability. For example, the binding of VEGF to VEGFR-2 leads to dimerization of the receptor, followed by intracellular activation of the PLCgamma;PKC-Raf kinase-MEK-mitogen-activated protein kinase (MAPK) pathway and subsequent initiation of DNA synthesis and cell growth, whereas activation of the phosphatidylinositol 3' -kinase (PI3K)-Akt pathway leads to increased endothelial-cell survival. Activation of PI3K, FAK, and p38 MAPK is implicated in cell migration signaling.

|  |  |  |
| --- | --- | --- |
| Reference pathway | 184% 150% 122% 100% 82% 67% 55% | 图片下载 |
